# Intestinal and esophageal microbiota in esophageal cancer development and treatment

**DOI:** 10.1080/19490976.2025.2505118

**Published:** 2025-05-16

**Authors:** Yuta Baba, Kohei Tajima, Kiyoshi Yoshimura

**Affiliations:** a Department of Clinical Immuno Oncology, Clinical Research Institute for Clinical Pharmacology and Therapeutics, Showa Medical University, Tokyo, Japan; b Division of Hematology, Department of Medicine, Showa Medical University Fujigaoka Hospital, Kanagawa, Japan; c Department of Gastroenterological Surgery, Tokai University School of Medicine, Kanagawa, Japan

**Keywords:** Gut microbiota, intestinal microbiota, esophageal microbiota, esophageal cancer, esophageal adenocarcinoma, esophageal squamous cell carcinoma

## Abstract

Esophageal cancer (EC) is the eleventh most commonly diagnosed cancer, and its prognosis remains poor. Several challenges remain for improving the clinical outcomes of EC, and improving technologies for early detection, diversifying treatment options, and advancing personalized treatment are essential. Alterations in the intestinal and esophageal microbiota are associated with the pathogenesis and progression of EC; for instance, *Fusobacterium nucleatum* is important in the pathogenesis and progression of esophageal squamous cell carcinoma. Therefore, a novel diagnostic biomarker may be identified using the intestinal microbiota. Furthermore, targeting the intestinal and esophageal microbiota may help in the early detection of EC, use of a novel prognostic biomarker, and even the detection of a therapeutic target, resulting in a more individualized therapeutic approach for EC. In this review, we summarize the clinical research focused on the intestinal and esophageal microbiota in EC development and its treatment, and discuss the challenges in the clinical application of intestinal and esophageal microbiota.

## GRAPHICAL ABSTRACT

**Figure:**
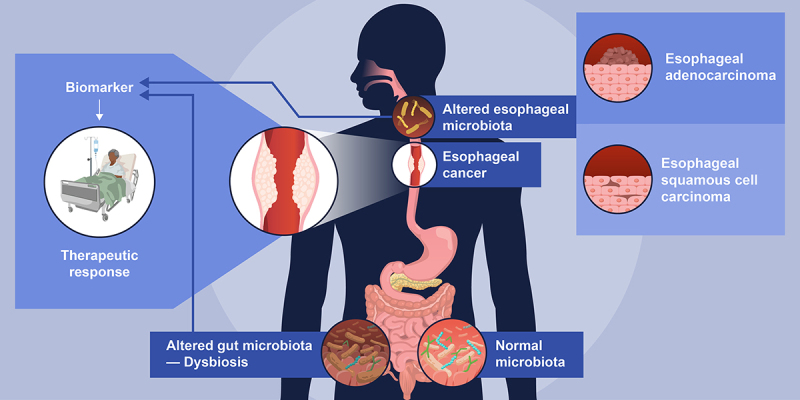

## Introduction

Esophageal cancer (EC) is the eleventh most commonly diagnosed cancer and the seventh leading cause of cancer-related deaths worldwide, with an estimated 510,716 new cases constituting 2.6% of all cancerous diagnoses and 445,129 deaths constituting 4.6% of all cancer-related deaths by 2022.^1^

Esophageal squamous cell carcinoma (ESCC) and esophageal adenocarcinoma (EAC) are EC subtypes. Approximately 90% of worldwide cases are ESCC, which is more widespread in East Asia, Southern Africa, East Africa, and Southern Europe.^2^ In contrast, EAC is more common in North America, Australia, and Western Europe,^3^ where its incidence has increased four-fold over the past four decades.^4,5^ Risk factors for ESCC include the consumption of tobacco, alcohol, pickled vegetables, hot foods, and poverty,^2^ whereas those for EAC include gastroesophageal reflux disease (GERD), Barrett’s esophagus (BE), obesity, and metabolic syndrome.^3^

The identification of these characteristics and risk factors has been well investigated. However, the projected number of cases of EC is expected to increase to 957,000 (141,300 EAC cases and 806,000 ESCC cases) by 2040, with deaths rising to 880,000.^6^ Several clinical challenges to improve clinical outcomes of EC remain. EC is often diagnosed at an advanced stage, and its early detection is difficult because patients only present with few symptoms in the early stage.^7^ Endoscopy is a well-established diagnostic procedure for EC. However, access to high-precision testing varies among different regions worldwide.^8,9^ The application of endoscopy is still limited due to its invasive nature and high cost. Endoscopy is effective for the early detection of EC, but its contribution to improving early detection is limited by challenges in performing screening over a wide area. Novel techniques such as the use of blood-based biomarkers for EC diagnosis are being developed.^10–13^ However, a standard method has not yet been established. Similarly, various predictive biomarkers for treatment efficacy are being developed via blood examination,^14,15^ imaging,^15^ and tumor tissue sampling.^16,17^ These biomarkers can be important indicators for determining optimal treatment strategies in individual patients. Nonetheless, their use in standard clinical settings should be further evaluated and validated. Several treatment strategies including surgery, radiation therapy, chemotherapy, and use of immune checkpoint inhibitors (ICIs) have been established. However, the efficacy of these therapies is often limited. Further, the risk of recurrence in patients with advanced-stage cancer who underwent surgery is high, and the treatment options for patients with recurrence are limited. To address these challenges, the methods used for early detection should be improved, and treatment options must be diversified. Moreover, individualized treatments should be developed.

To identify novel approaches, based on an increased understanding of the role of intestinal microbiota in humans, the importance of incorporating intestinal microbiota research into cancer studies is higher than ever. Recent studies have revealed that the intestinal microbiota can influence cancer progression, treatment response, and patient immunity. Esophageal microbiota refers to the presence of specific microbiota in the esophagus. Previous studies have revealed that the tumor microenvironment affects the tumor microbiota. Hence, the tumor microbiota plays an important role in the development and progression of EC.

Therefore, a novel diagnostic biomarker may be identified using the intestinal microbiota. Furthermore, targeting the intestinal and esophageal microbiota may help in the early detection of EC, use of a novel prognostic biomarker, and even the detection of a therapeutic target, resulting in a more individualized therapeutic approach for EC. Emphasizing the importance of intestinal and esophageal microbiota research may be essential in addressing the current clinical challenges associated with improving the clinical outcomes of EC. The current study aimed to summarize the clinical research focusing on the role of intestinal and esophageal microbiota on the development and treatment of EC.

### Gut microbiota and dysbiosis of the intestinal microbiota

The diverse human gut microbiota contains approximately 40 trillion microorganisms representing up to 1000 different bacterial species.^18,19^ The gut microbiota is often compared to “one organ” because intestinal bacteria have diverse effects on the physiological functions of the host via bacterial components and metabolites. The gut microbiota plays critical roles in the host; specifically, it influences immune maturation and homeostasis, cell proliferation, vascularization, neurological signaling, pathogenic burden, intestinal endocrine functions, bone density, and energy biogenesis; the biosynthesis of vitamins, steroid hormones, and neurotransmitters; and the metabolism of branched-chain and aromatic amino acids, dietary components, bile salts, drugs, and xenobiotics.^20,21^

The bacterial composition of the gut microbiota changes with age, and bacterial diversity expands rapidly in infancy and stabilizes in childhood; by preadolescence, the number of bacterial taxa and functional genes present in the gut microbiome is similar to that in adulthood.^22^ Shotgun metagenomics sequencing through random sequencing of all genes established that the intestinal microbiota of healthy human is dominated by phyla Firmicutes, Bacteroidetes, Actinobacteria, Proteobacteria and Verrucomicrobia. Phyla Firmicutes and Bacteroidetes represent 90% of the intestinal microbiota.^21^

The composition of the gut microbiota varies, and multiple endogenous and exogenous factors influence the gut microbiota.^23^ Dysbiosis is defined as the alteration or imbalance in the structure, composition, and function of the microbial communities.^24^ It has various interpretations, but in this review, the abovementioned definition is adopted because it fits the objectives and context. This definition requires considering that, due to the wide inter-individual variation of these communities, there is no gold standard for determining the ideal composition of healthy gut microbiota.

Dysbiosis of intestinal microbiota affects various diseases such as inflammatory bowel disease,^25^ auto-immune disease,^26^ obesity,^27^ type 2 diabetes,^28^ and allergy.^29^ Furthermore, dysbiosis is associated with various cancers, including colorectal cancer,^30^ gastric cancer,^31^ prostate cancer,^32^ breast cancer,^33^ acute myeloid leukemia,^34^ malignant lymphoma.^35^

### Association between dysbiosis of the intestinal microbiota in fecal samples and esophageal cancer

An increasing number of studies have focused on dysbiosis of intestinal microbiota in EC patients. Table 1 shows the previous studies investigating the association between the intestinal microbiota identified via fecal sample analysis and EC, mainly ESCC.^36–52^

**Table 1.** Studies on intestinal microbiota in esophageal cancer.

Author (year)	Participants	Objective	Country	Inclusion criteria for patients	Inclusion criteria for healthy controls	Exclusion criteria	Sampling approach for fecal samples	Diagnostic approaches	16S rRNA sequencing platform	Analysis methods	Results
Deng et al. ^36^	HC (*n* = 23), EC (*n* = 23)	Diagnosis of EC	China	Patients with newly diagnosed ESCC without prior medical treatment	Sex- and age-matched subjects	Chronic diarrhea or constipation, gastrointestinal disorders, use of probiotics, yogurt, traditional Chinese medicine, antibiotics, or other drugs influencing gut microbiota within the past month	Fresh stool	16S rRNA gene amplicon sequencing (V4)	Illumina MiSeq (San Diego, California)	LEfSe method	*Streptococcus* and *Bifidobacterium* were more abundant in EC, whereas those of *Lachnospira* and *Bacteroides* were less abundant compared with HC. Membrane transport, transcription, metabolism, xenobiotics biodegradation, metabolism, genetic information processing, and infectious disease pathways were enriched in EC.
Ishaq et al. ^37^	HC (*n* = 10), ESCC (*n* = 15)	Diagnosis of ESCC	China, Pakistan	Patients diagnosed with ESCC	Not available	Use of antibiotics, prebiotics, or probiotics within 30 days before sample collection	Fresh stool	16S rRNA gene amplicon sequencing (V3) qPCR	Illumina HiSeq 2500	*t* test	*Bacteroides* and *Escherichia-Shigella* were more abundant in ESCC, whereas *Prevotella* was less abundant in ESCC compared with HC.
Shen et al.^38^	HC (*n* = 17), ESCC (*n* = 21)	Diagnosis of ESCC	China	Patients with newly diagnosed ESCC without prior medical treatment	Subjects with similar dietary habits	Not available	Fresh stool	16S rRNA gene amplicon sequencing (V3–4)	Not available	Kruskal – Wallis test	*Enterococcus, Klebsiella* and *Streptococcus* were more abundant in ESCC, whereas *Fusobacterium* was less abundant in ESCC compared with HC.
Cheung et al. ^39^	HC (*n* = 16), ESCC (*n* = 15)	Diagnosis of ESCC	China	Patients with locally advanced or metastatic ESCC	Sex-matched healthy subjects >55 years old, no history of cancer, no probiotic, prebiotic, synbiotic, or antibiotic use within 1 month before sample collection	Acute gastrointestinal disorders, Use of antibiotics within 1 month, chemotherapy history within 2 months	Fresh stool	16S rRNA gene amplicon sequencing (V4)	Illumina MiSeq	Multinomial logistic regression	The log-ratios of *Streptococcus* to *Butyricicoccus* and *Streptococcus* to *Lachnospiraceae* NK4A136 group were higher in ESCC than in HC.
Li et al. ^40^	HC (*n* = 147), ESCC (*n* = 40)	Diagnosis of ESCC	China	Patients with locally advanced or stage IV ESCC, aged >18 yr, no operation or antitumor treatment within 6 months before enrollment, normal organ function, no probiotic, prebiotic, or antibiotic use within 2 weeks	Not available	Intestinal obstruction, Use of prebiotics, probiotics, or antibiotics within 2 weeks, unqualified or uncollectable baseline fecal samples	Fresh stool	16S rRNA gene amplicon sequencing (V4)	Illumina HiSeq 2500	LEfSe method, Wilcoxon rank-sum test	*Butyricicoccus pullicaecorum* was less abundant in ESCC compared with HC.
Xu et al. 41	ESCC (*n* = 46)	Prognosis of ESCC for ICIs	China	Patients newly diagnosed with thoracic ESCC (T2–4aNanyM0 or T1N1–3M0), aged 18–75 yr, with lesions located >20 cm from the incisors, PS score of 0 or 1, and at least one measurable lesion Treatments: Two cycles of camrelizumab combined with carboplatin and albumin paclitaxel before surgery	None	Lymph node metastases in supraclavicular/cervical regions, autoimmune or interstitial lung disease, receiving immunosuppressive therapy, high risk of esophageal perforation or active hemorrhage	Fresh stool Sampling time points^1^: within 3 days before neoadjuvant therapy,^2^ 3 days before surgery, and^3^ at the first postoperative defecation	16S rRNA gene amplicon sequencing (V3–4)	Illumina NovaSeq 6000	LEfSe method	The abundance of *Dialister* was higher in nonpathological complete response ESCC. *Pyramidobacter*, *Butyricimonas*, *Prevotella*, *Barnesiella*, and *Odoribacter* were higher in major pathological response ESCC.
Maruyama et al. ^42^	ESCC (*n* = 783)	Prognosis of ESCC for surgery	Japan	Patients diagnosed with ESCC scheduled for esophagectomy	None	Not available	Bacteriological sample collection media (Pro-Media FC-20; ELMEX, Tokyo, Japan), sampling within 1 week before esophagectomy	Culture-based	None	Univariate Cox proportional hazards model	*Bacillus* abundance was higher in favorable prognosis ESCC. *Proteus mirabilis* abundance was higher in favorable prognosis ESCC.
Hasuda et al. ^43^	HC (*n* = 10), ESCC (*n* = 21)	Diagnosis of ESCC	Japan	Patients diagnosed with ESCC scheduled for neoadjuvant therapy and thoracoscopic subtotal esophagectomy Treatments: 5-Fluorouracil plus cisplatin (FP), docetaxel plus cisplatin and 5-fluorouracil, or FP plus radiation	Subjects without serious medical history, regardless of smoking and drinking habits	Not available	Fresh stool Sampling time points^1^: before treatment,^2^ Day 5 of neoadjuvant therapy,^3^ after neoadjuvant therapy,^4^ 2 weeks after surgery, and^5^ 3 months after surgery	16S rRNA gene amplicon sequencing (V3–4)	Illumina MiSeq	LEfSe method	*Streptococcus* abundance was higher, whereas fecalibacterium abundance was lower in ESCC than in HC. *Streptococcus* increase and *Faecalibacterium* decrease were more pronounced postsurgery.
Sasaki et al. ^44^	ESCC (*n* = 51)	Prognosis of ESCC for CRT	Japan	Patients diagnosed with ESCC without prior treatment	None	Provide no informed consent, No defecation before treatment, or other malignancies	Stool collection kit containing guanidine (TechnoSuruga Laboratory, Shizuoka, Japan) Sampling time points^1^: within 1 week before treatment,^2^ within 3 weeks after chemoradiotherapy,^3^ within 2 weeks after two cycles of systemic chemotherapy and neoadjuvant chemotherapy, and^4^ within 1 month after surgery	16S rRNA gene amplicon sequencing	Ion Torrent Personal Genome Machine (Thermo Fisher Scientific, Waltham, Massachusetts)	LEfSe method	The relative abundance of Lactobacillaceae at the family level was higher in ESCC patients with partial or complete response.
Huang et al. ^45^	HC (*n* = 50), ESCC (*n* = 50)	Diagnosis of ESCC	China	Patients newly diagnosed with ESCC, aged 40–80 yr	Ethnicity, sex, and age (±5 yr) matched subjects	Use of antibiotics within 3 months before the study, other organ tumors, history of gastrointestinal diseases, or gastroesophageal-related surgeries	Fresh stool	16S rRNA gene amplicon sequencing (V3–4) and untargeted metabolomics analyses (LC-MS)	Illumina NovaSeq	LEfSe method, Wilcoxon rank-sum test	The abundance of *Fusobacteriaceae* and *Lactobacillus* were higher in ESCC than in HC. The level of Gibberellin A34 was elevated, whereas 12-hydroxydodecanoic acid was reduced in ESCC compared with HC.
Gao et al. ^46^	HC (*n* = 20), ESCC (*n* = 20)	Diagnosis of ESCC	China	Patients diagnosed with ESCC	Sex, age, body mass index, and dietary habits matched	Gastrointestinal inflammation within 8 weeks, recent use of antibiotics or immunosuppressive drug use, prior chemotherapy, radiotherapy, or immunotherapy, digestive system diseases, metabolic disorders, and other malignancies	Fresh stool	16S rRNA gene amplicon sequencing (V3–4) and untargeted metabolomics analyses (LC-MS)	Illumina NovaSeq	LEfSe method, *t* test	The abundance of *Bacteroides stercoris* and *Prevotella copri* were higher in ESCC than in HC. The relative abundances of indoles and their derivatives, tropane alkaloids, lipids, and lipid-like molecules were higher in ESCC than in HC. *Phenylethanolamine* and *despropionyl p-fluoro fentanyl* could serve as reliable biomarkers to differentiate ESCC from HC.
Otsuka et al. ^47^	ESCC (*n* = 51)	Prognosis of ESCC for surgery	Japan	Patients diagnosed with ESCC with tumor stage lower than T4b scheduled for neoadjuvant chemotherapy and thoracoscopic resection	None	Not available	Stool collection kit containing guanidine (TechnoSuruga Laboratory, Shizuoka, Japan) Sampling time point: within 3 weeks before surgery	16S rRNA gene amplicon sequencing (V3–4)	Illumina MiSeq	Mann – Whitney *U* test	The abundance of *Butyricimonas* and *Actinomyces* were higher in recurrent ESCC.
Wu et al. ^48^	ESCC (*n* = 30), HC (*n* = 30)	Diagnosis of ESCC	China	Patients diagnosed with primary EC who had not undergone radiotherapy, chemotherapy, or surgery, aged >18 yr	With normal bowel habits	Diabetes or depression, use of antibiotics, H2 receptor antagonists, proton pump inhibitors, or probiotics within the past month, and with other cancers or dental bacterial diseases	Fresh stool	16S rRNA gene amplicon sequencing (V3–4)	Illumina NovaSeq 6000	LEfSe method	The abundance of *Bacteroides* was higher in ESCC than in HC. The abundance of *Bacteroides* increased with ESCC progression.
van den Ende et al. ^49^	EAC (*n* = 150), ESCC (*n* = 22)	Prognosis of EC for neoadjuvant and surgery	Netherlands	Patients diagnosed with resectable stage II – III EAC or ESCC scheduled for neoadjuvant chemoradiotherapy or resectable stage II – III EAC scheduled for neoadjuvant chemoradiotherapy combined with ICIs	None	Not available	Feces tubes (Sarstedt Inc, Nümbrecht, Germany) Sampling time points: baseline, during treatment (Week 3 of neoadjuvant chemoradiotherapy), and before surgery	16S rRNA gene amplicon sequencing (V3–4)	Illumina MiSeq	DESeq2	The abundance of *Desulfovibrio*, *Subdoligranulum*, and *Parabacteroides* was higher, whereas the abundances of *Bifidobacterium*, *Phascolarctobacterium*, and *Escherichia coli* or *Shigella* were lower in the pathological complete response of EC than at Week 3 and before surgery. The abundances of *Phascolarctobacterium*, *Bacteroides*, and *Bifidobacterium* were higher, whereas the abundances of *Kosokonia*, *Romboutsia*, and *Lactobacillus* were lower in shorter progression-free survival of EC than at Week 3 and before surgery.
Li et al. ^50^	ESCC (*n* = 31)	Prognosis of ESCC for chemotherapy	China	Patients newly diagnosed with ESCC, confirmed as locally advanced or metastatic, residing in northern China, and maintained an oral diet Treatments: Paclitaxel and cisplatin	None	Bowel obstruction, infection, inflammatory bowel disease, chronic disease, use of antibiotics or probiotics within 1 month before fecal sample collection, or with a long-term medication history	MGIEasy collecting tube	16S rRNA gene amplicon sequencing (V4)	Illumina HiSeq 2500	Wilcoxon rank-sum test	The abundance of *Bacteroides plebeius* and *Bacteroides ovatus* were higher in chemotherapy responders.
Shaikh et al. ^51^	EAC (*n* = 20), ESCC (*n* = 3)	Prognosis of EC for neoadjuvant ICIs plus CRT	USA	Patients diagnosed with stage II – III resectable EC Treatments: Neoadjuvant ICIs (nivolumab or nivolumab/relatlimab) plus CRT (weekly carboplatin/paclitaxel and concurrent radiation) before surgical resection	None	Not available	Fresh stool or OMNIgene GUT (DNA Genotek, Stittsville, Ontario, Canada, OM-200) Sampling time point: within the first 42 days of treatment	16S rRNA gene amplicon sequencing (V1–2), metabolomics analyses	Not available	LEfSe method, Mann – Whitney *U* test	The abundance of *Ruminococcus callidus*, *Fusicatenibacter saccharivorans*, *and Roseburia inulinivorans* was higher in the pathological complete response of EC. The relative abundances of sphingolipid and primary bile acids were higher in the pathological complete response of EC.
Liu et al. ^52^	ESCC (*n* = 68), HC (*n* = 19)	Diagnosis of ESCC and prognosis of EC for neoadjuvant immunochemotherapy	China	Patients diagnosed with ESCC undergoing neoadjuvant immunochemotherapy Treatments: Neoadjuvant immunochemotherapy involves platinum combined with paclitaxel and pembrolizumab, camrelizumab, tislelizumab, toripalimab, nivolumab, or sintilimab before curative esophagectomy and lymphadenectomy	Without any malignancies by clinical evaluation Living in the same household as the ESCC patients	Recent use of antibiotics, prebiotics, probiotics, steroids, or immunosuppressants within 1 month before fecal sample collection	Fresh stool Sampling time points^1^: baseline (before neoadjuvant immunochemotherapy) and^2^ within a 3-day window following completion of neoadjuvant immunochemotherapy	16S rRNA gene amplicon sequencing (V3–4)	Illumina NovaSeq 6000	LEfSe method, Mann – Whitney *U* test	The abundances of *Haemophilus*, *Phascolarctobacterium*, *Fusobacterium*, and *Escherichia shigella* were higher in ESCC, whereas those of *Faecalibacterium*, *UCG 002*, *Eubacterium ventriosum* group, *Firmicutes unclassified*, *Cuneatibacter*, and *Christensenellaceae R 7* group were lower in ESCC than in HC. The abundances of *Anaerostipes*, *Erysipelotrichaceae UCG 003*, *Subdoligranulum*, *Faecalibacterium*, *Eubacterium eligens* group, and *Phascolarctobacterium faecium* were higher in the responder of neoadjuvant immunochemotherapy, whereas those of *Enterobacteriaceae*, *Corynebacteriaceae*, *Veillonella*, *Corynebacterium*, *Streptococcus parasanguinis*, and *Prevotella buccalis* were higher in the nonresponder.

CRT, chemoradiotherapy; EAC, esophageal adenocarcinoma; EC, esophageal cancer; ESCC, esophageal squamous cell carcinoma; HC, healthy control; ICIs, immune checkpoint inhibitors; LEfSe, linear discriminant analysis effect size.

**Figure 1.**
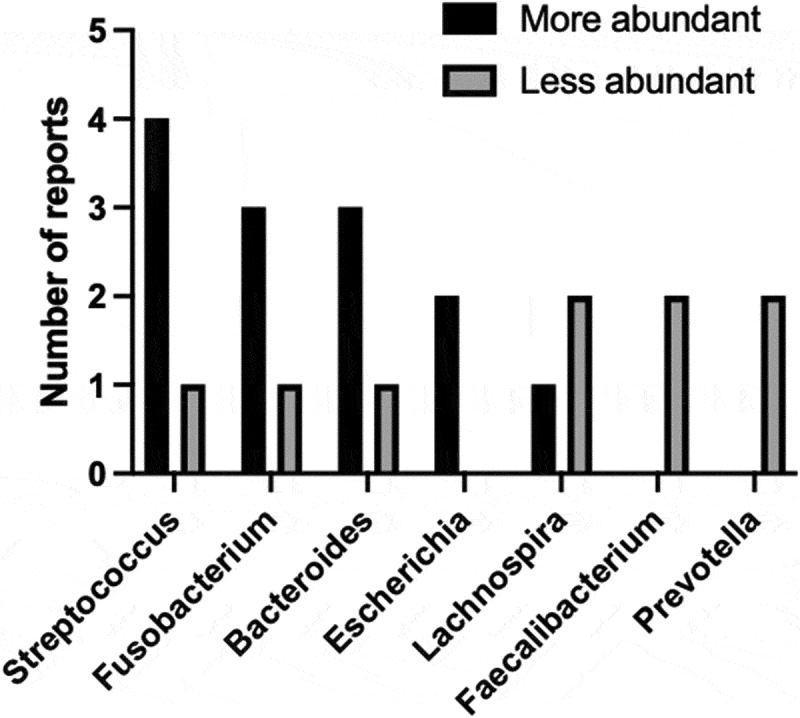
The bacterial genera reported to be more or less abundant in the intestinal microbiota of fecal samples from patients with esophageal squamous cell carcinoma compared to healthy subjects.

The findings across the studies have consistently shown that patients with ESCC exhibited altered intestinal microbiota profiles in fecal samples compared with healthy controls. In particular, in each study, the number of specific bacterial genera or species in patients with ESCC either increased or decreased compared with that in healthy controls.^36–40,43,45,46,48,52^ The bacterial genera that are reported to be more or less abundant in the intestinal microbiota of fecal samples from patients with ESCC compared to healthy subjects in two or more papers are summarized in Figure 1. Notably, the common findings include the following: Patients with ESCC were more likely to have a higher abundance of *Streptococcus*,^28,36,39,43^ *Fusobacterium*,^45,49,52^ and *Bacteroides*^37,46,48^ than healthy controls. Moreover, patients with ESCC were more likely to have a lower abundance of *Lachnospira*,^36,39^ *Prevotella*,^37,41^ and *Faecalibacterium*^43,52^ than healthy controls. These variations in the abundance of specific bacterial species attempt to use as biomarkers for diagnosing ESCC that they can be detected noninvasively and cost-effectively. However, the intestinal microbiota that characterize ESCC has been inconsistent. Similarly, regarding diversity in patients with ESCC compared to healthy controls, the findings were inconsistent.

The bacterial genera reported in two or more papers are extracted. Details are described in Table 1.

Moreover, previous studies have revealed that the intestinal microbiota can be used as a predictive biomarker for therapeutic response in patients with ESCC.^41,42,44,47,49–52^ The intestinal microbiota in fecal samples may predict the response of ICIs in patients with ESCC,^41,52^ similar to those with other types of cancers. Responders to neoadjuvant therapy combined with ICIs for ESCC had a high abundance of *Pyramidobacter*, *Butyricimonas*, *Prevotella*, *Barnesiella*, *Odoribacter*,^41^ *Anaerostipes, Erysipelotrichaceae UCG 003, Subdoligranulum, Faecalibacterium, Eubacterium eligens group*,and *Phascolarctobacterium faecium*.^52^ Meanwhile, non-responders had a high abundance of *Dialister*,^41^ *Enterobacteriaceae*, *Corynebacteriaceae*, *Veillonella*, *Corynebacterium*, *Streptococcus parasanguinis*, and *Prevotella buccalis*.^52^ In addition, patients with higher levels of *Bacteroides plebeius* and *Bacteroides ovatus* were more responsive to chemotherapy.^50^ Patients with recurrence requiring surgery had high levels of *Butyricimonas* and *Actinomyces*.^47^ Based on these results, the abundance of specific bacterial species can play an important role in pretreatment monitoring. However, the intestinal microbiota profiles that are used as predictive markers of therapeutic response in ESCC are inconsistent.

There are only a few reports on the association between EAC and intestinal microbiota in fecal samples. In particular, they are only limited to studies that analyzed EAC and ESCC together, as shown in Table 1.^36,49,51^ The abundance of *Bacteroides*, *Flavonifractor*, *Incertae sedis*, *Odoribacter*, *Parabacteroides*, and *Parasutterella* in rectal samples (sampling method not stated) differed between healthy controls and patients with EAC.^53^

These intestinal microbiota specifically affects the physiological functions of the hosts. The dysbiosis of the intestinal microbiota changes the metabolites, affecting the metabolic pathways, inflammation, and immune function and has attracted attention for its possible role in the development and pathogenesis of ESCC. Patients with ESCC exhibited a significant increase in the community richness of intestinal microbiota in fecal samples. Further, in patients with ESCC, the abundance of SCFA-producing, particularly butyrate-producing, bacteria decreased. Meanwhile, the abundance of LPS-producing bacteria increased. These phenomena may lead to the destruction of the intestinal barrier and the promotion of the intestinal permeability and inflammation. In addition, inflammatory factors and LPS may then be released into the bloodstream to target organs via long-distance migration, and such long-term effects of chronic exposure contribute to the development of ESCC.^36,39^ Furthermore, differential metabolites between patients with ESCC and healthy controls, mainly amino acids, peptides, and analogs on nontargeted metabolomics analyses, significantly inhibited multiple amino acid metabolism pathways in patients with ESCC patients via pathway analysis of the microbiota and metabolites. Therefore, these patients had significant disruption in amino acid metabolism, which could possibly affect the development of ESCC.^45^ An increase in the number of *Enterobacteriaceae* and *Lactobacillus* significantly reduced the L-aspartate and pantothenic acid levels, which may be involved in the development of ESCC by downregulating the protein expressions in the pantothenate and coenzyme A biosynthesis pathways.^45^ Similarly, according to nontargeted metabolomics analyses, patients with ESCC had higher relative abundance of differential metabolites, such as indoles and derivatives, tropane alkaloids, lipids, and lipid-like molecules, than healthy controls.^46^ By metagenome function prediction, unsaturated fatty acids, ascorbate, and Aldrete metabolism and the hypoxia-inducible factor 1 signaling pathway were significantly associated with differential metabolites.^46^ *Prevotella* were significantly positively correlated with indoles and their derivatives, therefore it could influence the occurrence of ESCC through the regulation of indoles and derivatives.^46^ Based on these results, the dysbiosis of the intestinal microbiota changes the metabolites, and it may affect the development of ESCC by regulating multiple metabolic pathways. The intestinal microbiota and its metabolites in fecal samples are being integrated for use as biomarkers for diagnosing ESCC.^45,46^

### Esophageal microbiota

The presence of the esophageal microbiota has been revealed, and its relationship with various esophageal diseases has been investigated as well as intestinal microbiota.

In 1983, a pioneering study of esophageal microbiota using culture techniques identified *Streptococcus viridans*, *Haemophilus influenzae, Neisseria catarrhalis, Streptococcus group B, Streptococcus faecalis*, and *Klebsiella pneumoniae* as commensals populating the esophageal microbiota of healthy subjects.^54^

In 2004, using broad-range 16S rDNA PCR, 95 bacterial species from six phyla – Firmicutes, Bacteroidetes, Actinobacteria, Proteobacteria, Fusobacteria, and TM7 – were identified in the esophageal microbiota.^55^ At the genus level, *Streptococcus*, *Prevotella*, and *Veillonella* were the most common bacteria within the esophageal microbiota.^55^ A comparison of the microbiota in the lower esophagus, upper esophagus, and oral mucosa of healthy subjects revealed comparable compositions among them.^56^ The esophageal microbiota was classified into two types: type I is dominated by Streptococcus and concentrated in the phenotypically normal esophagus, while type II contains a greater proportion of gram-negative anaerobes or microaerophiles and is primarily correlated with esophagitis.^57^ Moreover, using 16S rRNA amplicon sequencing and shotgun sequencing, the esophageal microbiota was clustered into functionally distinct community types (esotypes) defined by the relative abundances of *Streptococcus* and *Prevotella*; the cluster dominated by *Streptococcus* (*Streptococcus mitis/oralis/pneumoniae*) was enriched for the pentose phosphate pathway as well as fructose and mannose metabolism that dominated by *Prevotella* (*Prevotella melaninogenica* and *Prevotella pallens*) was enriched for lipopolysaccharide biosynthesis, and the other cluster was the intermediate type with respect to *Streptococcus* and *Prevotella* but had increased levels of *Haemophilus parainfluenzae* and *Rothia mucilaginosa* for the glycolysis as well as the pathways involved in the metabolism of SCFA.^58^

Then, recent studies revealed alterations in the esophageal microbiota of patients with EC.

### Association between esophageal microbiota and esophageal adenocarcinoma

Table 2 depicts the studies investigating the association between esophageal microbiota and EAC.^49,53,59–67^

**Table 2.** Esophageal microbiota in esophageal adenocarcinoma.

Author (year)	Participants	Objective	Country	Inclusion criteria for patients	Inclusion criteria for healthy controls	Exclusion criteria	Sampling approach	Diagnostic approaches	16S rRNA sequencing platform	Analysis methods	Results
Elliott et al. ^59^	HC (*n* = 20), NDBE (*n* = 24), HGD (*n* = 23), EAC (*n* = 19)	Microbiota characterization in EAC	United Kingdom	Patients who had an endoscopic and histological diagnosis and aged 20–90 yr	Subjects with normal endoscopy and aged 20–90 yr	Current infection, recent antibiotic treatment, previous chemotherapy, or unrelated pathological findings	Endoscopic brushing (normal squamous esophagus), endoscopic biopsies (BE and normal squamous esophagus), and endoscopic mucosal resection and surgical specimens	16S rRNA gene amplicon sequencing (V1–2)	Illumina MiSeq	LEfSe method	Significant differences in microbial community structure in EAC tissues; modest reduction in diversity in BE. The abundance of *Lactobacillus* and *Streptococcus* was higher in EAC tissues than in the healthy controls and BE. No bacteria were identified that discriminated between healthy controls and BE or between BE and EAC.
Snider et al. ^60^	HC (*n* = 16), NDBE (*n* = 14), LGD (*n* = 6), HGD (*n* = 5), EAC (*n* = 4)	Microbiota characterization in EAC	USA	Endoscopic and histological diagnosis, histologically confirmed BE ≥2 cm, and no prior endoscopic therapy	No prior BE history, proton pump inhibitor use at least once daily, or no acid suppression in the prior month	Not available	Endoscopic brushing (no nodules, masses, or focal lesions) HC: Gastric cardia that is within 1 cm of the squamocolumnar junction Patients: BE tissue	16S rRNA gene amplicon sequencing (V4)	Not available	LEfSe method	The abundance of *Enterobacteriaceae* and *Verrucomicrobiaceae*, specifically *Akkermansia muciniphila*, was higher, whereas the abundance of *Veillonella* was lower in nonfocal lesions of HGD or EAC than in BE or LGD.
Li et al. ^61^	HC (*n* = 16), EAC (*n* = 11)	Microbiota characterization in EAC	China	Endoscopic and histological diagnosis	No digestive symptoms or esophagogastric mucosal lesions as confirmed by endoscopy	Systemic infections, other malignancies, preoperative neoadjuvant chemoradiotherapy, biotherapy, history of gastrointestinal surgery, antibiotic use, or microecological preparations within 2 months	Endoscopic biopsies HC: Normal esophageal specimens (upper, middle, and lower segments) Patients: Tumor tissues	16S rRNA gene amplicon sequencing (V3–4)	Illumina MiSeq	LEfSe method, *t* test, Wilcoxon rank-sum test	At the phylum level, the relative abundance of *Firmicutes* was higher, and that of *Proteobacteria* was lower in the EAC tissues than in the HC.
Lopetuso et al. ^62^	HC (*n* = 10), BE (*n* = 10), EAC (*n* = 6)	Microbiota characterization in EAC	Italy	Endoscopic and histological diagnosis	Not available	Previous endoscopic or surgical treatment, active oral cavity infection, hepatitis B or C virus or human immunodeficiency virus infection, anticoagulation therapy, inability to undergo upper endoscopy, recent use of antibiotics, probiotics, proton pump inhibitor, or histamine 2 receptor antagonist in the 2 months	Endoscopic biopsies HC: Two biopsies from normal esophageal mucosa Patients: BE, two biopsies from metaplastic lesions and two from normal esophageal mucosa EAC, two biopsies from neoplastic lesions	16S rRNA gene amplicon sequencing (V3–4)	Illumina MiSeq	LEfSe method, Mann – Whitney *U* test	The abundances of *Prevotella*, *Veillonella*, and *Leptotrichia* were increased, and that of *Streptococcus* was reduced in EAC tissues compared with HC.
Zhou et al. ^63^	HC (*n* = 16), GERD (*n* = 11), RE (*n* = 20), BE (*n* = 17), EAC (*n* = 6)	Microbiota characterization in EAC	Australia	Endoscopic and histological diagnosis and aged >18 yr	Referred for endoscopy due to iron deficiency anemia or lower abdominal pain with no endoscopic evidence of esophageal, gastric, or duodenal diseases	Use of antibiotics or probiotics within 3 months	Endoscopic brushing (three repeat brushings at target sites) and endoscopic biopsies (1 cm above the squamocolumnar junction or at the pathology site)	16S rRNA gene amplicon sequencing (V1–3), unbiased quantitative proteomics analysis	Illumina MiSeq	Mann – Whitney *U* test	The EAC was characterized by a shift toward *Firmicutes*, mainly *Staphylococcus aureus*, *Streptococcus infantis*, *Moryella* sp., and *Lactobacillus salivarius* as well as Proteobacteria but away from *Rothia mucilaginosa* relative to that in the HC. The BE and EAC were characterized by reduced expression of gastrointestinal epithelium markers keratin 13 and keratin 20 and increased expression of epithelial-to-mesenchymal transition markers vimentin and desmin compared with those in the HC.
Peter et al. ^64^	HC (*n* = 12), intestinal metaplasia (*n* = 9), LGD (*n* = 12), HGD (*n* = 10), EAC (*n* = 10)	Microbiota characterization in EAC	USA	Endoscopic and histological diagnosis	No evidence of BE	History of acute reflux symptoms, acute or chronic vomiting, and recent use of antibiotics or nonsteroidal antiinflammatory drug within the preceding 4 weeks	Endoscopic biopsies Two biopsies from each site, including the BE/EAC and visibly unaffected esophagus (3-cm cephalad)	16S rRNA gene amplicon sequencing (V4)	Illumina MiSeq	Mann – Whitney *U* test, Kruskal – Wallis test	The abundances of *Planctomyces*, *Nitrosopumilus*, and *Balneola* were reduced in EAC tissues.
Hao et al. ^53^	HC (*n* = 27), GERD (*n* = 37), BE (*n* = 32), EAC (*n* = 19)	Microbiota characterization in EAC	USA	Endoscopic and histological diagnosis	No reflux symptoms, normal pH range (fraction time of pH < 4 and DeMeester score < 14.72) on a 48-h pH monitoring test, and negative for endoscopic and histological esophagitis	Use of antibiotics within 2 weeks	Endoscopic brushing (10 times, the distal esophagus at approximately 3 cm above the gastroesophageal junction)	16S rRNA gene amplicon sequencing (V3–4)	Roche 454 FLX (Roche, Branford, Connecticut)	Multivariable linear regression analysis, Spearman’s rank correlation	Esophageal microbiota exhibited increased diversity along the GERD-BE-EA sequence. *Streptococcus* abundance decreased progressively, whereas *Atopobium*, *Actinomyces*, *Veillonella*, *Ralstonia*, *Burkholderia*, and *Lautropia* increased. Functional gene analysis revealed progressive enrichment of genes associated with antibiotic resistance, Toll-like and NOD-like receptor ligands, the nitrate-nitrite-nitric oxide pathway, and acetaldehyde metabolism.
Zaramella et al. ^65^	BE (*n* = 57), LGD (*n* = 8), HGD (*n* = 8), EAC (*n* = 7)	Microbiota characterization in EAC	Italy	Endoscopic and histological diagnosis	None	Presence of other cancer types, use of antibiotics/probiotics within 3 months, prior esophageal surgery	Targeted endoscopic biopsies from the distal esophagus	16S rRNA gene amplicon sequencing (V3–4)	Illumina MiSeq	Mann – Whitney *U* test	The abundances of *Bergeyella* and *Allopreovetell* were higher, and those of *Acinetobacter* and *Massilia* were lower in dysplastic BE or EAC than in non-dysplastic BE.
Greathouse et al. ^66^	EAC (*n* = 74), ESCC (*n* = 17)	Microbiota characterization in EC	USA	Endoscopic and histological diagnosis	None	Not available	Surgical specimens	16S rRNA gene amplicon sequencing (V3–4)	Illumina MiSeq	Mann – Whitney *U* test	The abundances of *Campylobacter*, *Fusobacterium*, *Prevotella*, and *Streptococcus* were higher in EC tissues than in the non-EC tissues.
van den Ende et al. ^49^	EAC (*n* = 40)	Prognosis of EAC for neoadjuvant ICIs plus CRT and surgery	Netherlands	Patients diagnosed with resectable stage II – III EAC or ESCC scheduled for neoadjuvant chemoradiotherapy and patients with resectable stage II – III EAC scheduled for neoadjuvant chemoradiotherapy combined with ICIs	None	Not available	Endoscopic biopsies from tumor tissue at baseline and during treatment	16S rRNA gene amplicon sequencing (V3–4)	Illumina MiSeq	DEseq2	The abundance of *Fusobacterium* was enriched and that of *Streptococcus* was reduced in EAC tissues of cases with shorter progression-free survival.
Shijimaya et al. ^67^	BE (*n* = 44), EAC (*n* = 21)	Microbiota characterization in EAC	Japan	Endoscopic and histological diagnosis	None	Not available	BE patients: Endoscopic biopsies obtained from the most proximal site of columnar-lined epithelium. EAC patients: Endoscopic biopsies taken from both EAC and non-EAC tissues at least >3 mm away from the cancerous area.	16S rRNA gene amplicon sequencing (V3–4)	Illumina MiSeq	Mann – Whitney *U* test	The abundances of *Actinomyces*, *Prevotella*, and *Anaerosinus* were lower in EAC than in BE.

BE, Barrett’s esophagus; EAC, esophageal adenocarcinoma; GERD, gastroesophageal reflux disease; HC, healthy controls; HGD, high-grade dysplasia; ICIs, immune checkpoint inhibitors; LEfSe, linear discriminant analysis effect size; LGD, low-grade dysplasia; NDBE, BE without dysplasia; RE, reflux esophagitis.

The findings across the studies consistently showed that the esophageal microbiota profiles in EAC tissues were altered compared with those in non-EAC or healthy tissues. In particular, in each study, the number of specific bacterial genera or species either increased or decreased.^53,59–67^ However, the esophageal microbiota that characterize EAC has been inconsistent. Similarly, the diversity of esophageal microbiota between patients with EAC and healthy controls is inconsistent. In addition, previous studies on the utility of the esophageal microbiota as a predictive biomarker of therapeutic response in patients with EAC were limited. For example, EAC tissues exhibited enriched *Fusobacterium* and reduced *Streptococcus* in patients with shorter survivals from neoadjuvant therapy to surgery.^49^

In relation to the EAC and esophageal microbiota, previous studies have emphasized the dynamic changes in the esophageal microbiota during the EAC cascade, which showed the progression from GERD to BE and finally to EAC. The esophageal microbiota differed between healthy controls and patients with GERD, BE, and EAC in the nonguided culture-based study.^68^ The predominant organisms shifted from gram-positive bacteria to gram-negative anaerobic bacteria in the EAC cascade. However, the esophageal microbiota of EAC is less well defined than that of GERD and BE. The increase in the abundance of *Veillonella* and the decrease in the abundance of *Streptococcus* occurred progressively along with the EAC cascade.^53,62^ The number of *Leptotrichia* increases in patients with BE and EAC. Then, a corresponding elevation in the number of *Veillonella* and *Prevotell* is observed. Thus, they could be plausible prooncogenic partners.^62^ The keystone genera, including *Atopobium*, *Veillonella*, *Burkholderia*, *Ralstonia*, and *Lautropia*, increase from the GERD and BE to the EAC sequence.^53^ Thus, these organisms can be potential biomarkers for monitoring progression.

The functional analysis can help provide an understanding of role of the esophageal microbiota in the development and progression of EAC. Alterations in the composition and abundance of the esophageal microbiota could promote the development of GERD, BE, and EAC via multiple mechanisms. The altered esophageal microbiota activated and stimulated multiple toll-like receptors (TLRs),^69–71^ inducible nitric oxide synthase,^72^ and nod-like receptor protein 3 (NLRP3),^71^ which then promoted malignant cellular behavior. TLR4 activation led to the activation of the nuclear factor kappa-light-chain-enhancer of activated B cells (NF-κB)^73^ and increased the expression of cyclooxygenase-2.^70^ Alterations in the esophageal microbiota lead to chronic inflammation and a decreased immune response, thereby establishing a microenvironment that is suitable for malignant transformation. In terms of other mechanism, the genes related to nitrate – nitrite – nitric oxide pathways were enriched along from the GERD and BE to the EAC sequence.^53^ The number of gram-negative bacteria increased in the esophageal microbiota of patients with EAC. This phenomenon may stimulate the expression of inducible nitric oxide synthase (iNOS). This can relax the lower esophageal sphincter and induce GERD. In addition, high concentrations of NO produced by iNOS can generate free radicals, causing DNA damage.^74^ NO and iNOS activities induce apoptosis, angiogenesis, and DNA damage during tumorigenesis.^75^ NO increases the expression of matrix metalloproteinase and its inhibitor and promotes the progression of dysplastic lesions in BE to invasive carcinoma.^72^ These functional changes are essential because they identify the pathways that could be involved in promoting tumorigenesis.

### Association between esophageal microbiota and esophageal squamous cell carcinoma

Table 3 shows the studies investigating the association between esophageal microbiota and ESCC.^61,76–101^

**Table 3.** Esophageal microbiota in esophageal squamous cell carcinoma.

Author (year)	Participants	Objective	Country	Inclusion criteria for patients	Inclusion criteria for healthy controls	Exclusion criteria	Sampling approach	Diagnostic approaches	16S rRNA sequencing platform	Analysis methods	Results
Gao et al. ^76^	HC (*n* = 30), ESCC (*n* = 100)	Characteristic in ESCC and prognosis of ESCC	China	Patients with ESCC who underwent esophagectomy	Age- and sex-matched subjects	Not available	Endoscopic biopsies (HC), surgical specimens (Patients: Tumor tissues and paired nontumor tissues located 3 cm distant to the tumor tissue)	Immunohistochemical detection and qPCR	None	Chi-square test, Cox proportional hazards regression modellog-rank test	*Porphyromonas gingivalis* was more prevalent in ESCC tissues than in adjacent nontumor tissues. Its presence correlated with poor differentiation, lymph node metastasis, advanced TNM stage, and poor overall survival.
Yamamura et al. ^77^	ESCC (*n* = 300), EAC (*n* = 12), others (*n* = 13)	Characteristic in EC and prognosis of ESCC	Japan	Patients with ESCC who underwent esophagectomy	None	Not available	Surgical specimens	qPCR	None	Chi-square test, log-rank test, Cox proportional hazards regression model	*Fusobacterium nucleatum* was more prevalent in EC tissues than in the nontumor tissues. *Fusobacterium nucleatum* in ESCC tissues was correlated with tumor stage and poor prognosis. *Fusobacterium nucleatum* contributes to the acquisition of aggressive tumor behavior through chemokine activation.
Liu et al. ^78^	ESCC (*n* = 45)	Characteristic in ESCC and prognosis of ESCC	China	Patients with ESCC who underwent esophagectomy	None	Use of antibiotics or micro-ecologics for at least 2 months, received neoadjuvant therapy, organic cardiovascular diseases, infection, reflux esophagitis, or recurrent EC	Surgical specimens	16S rRNA gene amplicon sequencing (V3–4)	Illumina MiSeq	*t* test, log-rank test, Cox proportional hazards regression model	The abundance of *Streptococcus* and *Prevotella* increased as the tumor stage increased. Combined *Streptococcus* and *Prevotella* abundance indicated unfavorable survival.
Shao et al. ^79^	ESCC (*n* = 67)	Characteristic in ESCC	China	Patients with ESCC who underwent esophagectomy	None	Not available	Surgical specimens (tumor and paired nontumor tissues ≥4 cm distant)	16S rRNA gene amplicon sequencing (V4)	Illumina MiniSeq	LEfSe method, Kruskal – Wallis test	*Fusobacterium* was more abundant, whereas *Streptococcus* was less contained in ESCC tissues than in nontumor tissues. The abundance of *Fusobacterium* increased as the tumor stage increased.
Yamamura et al. ^80^	ESCC (*n* = 551)	Characteristic in ESCC and prognosis of ESCC	Japan	Patients with ESCC who underwent esophagectomy	None	Not available	Surgical specimens (no definition of paired nontumor tissue)	qPCR	None	Chi-square test, log-rank test, Cox proportional hazards regression model	*Fusobacterium nucleatum* was more prevalent in ESCC tissues than in nontumor tissues. The abundance of *Fusobacterium* increased as the tumor stage increased. *Fusobacterium nucleatum* was correlated with poor recurrence-free survival and poor response to neoadjuvant chemotherapy.
Li et al. ^61^	HC (*n* = 16), ESCC (*n* = 17)	Characteristic in ESCC	China	Patients who had endoscopic and histological diagnosis	No digestive symptoms and esophagogastric mucosal lesions as confirmed by endoscopy	Systemic infections, other coexisting malignant tumors, preoperative neoadjuvant chemoradiotherapy, biotherapy, history of gastrointestinal surgery, use of antibiotics, or microecological preparations within 2 months	Endoscopic biopsies HC: Normal esophageal specimens (upper, middle, and lower segments) Patients: Tumor tissues	16S rRNA gene amplicon sequencing (V3–4)	Illumina MiSeq	LEfSe method, *t* test, Wilcoxon rank-sum test	At the phylum level, the relative abundance of Fusobacteria was higher, and that of Actinobacteria was lower in ESCC tissues than in HC.
Li et al. ^81^	HC (*n* = 70), esophagitis (*n* = 70), LGIN (*n* = 70), HGIN (*n* = 19), ESCC (*n* = 7)	Characteristic in ESCC	China	Patients who had endoscopic and histological diagnosis	Not available	Not available	Endoscopic brushing (five looping brushes at the lesion or normal middle esophagus), endoscopic biopsies (at the brush sampling site)	16S rRNA gene amplicon sequencing (V4)	Illumina MiniSeq	Wilcoxon rank-sum test, Kruskal – Wallis test	*Neisseria*, *Haemophilus*, and *Porphyromonas* were more abundant, whereas *Streptococcus* was less contained in ESCC tissues than in HC. *Neisseria*, *Haemophilus*, and *Porphyromonas* were increased, whereas *Streptococcus* showed a decreasing tendency with ESCC progression.
Kovaleva et al. ^82^	ESCC (*n* = 48)	Characteristic in ESCC	Russia	Patients with ESCC who underwent esophagectomy	None	Patients who lived <2 months from the date of surgery	Surgical specimens (histologically verified tumor tissue and paired nontumor tissue that normal esophageal tissue located as far as possible from the tumor)	16S rRNA gene amplicon sequencing (V3–4)	Illumina MiSeq	Mann – Whitney *U* test	*Staphylococcus* was more abundant in ESCC tissues than in nontumor tissues.
Li et al. ^83^	HC (*n* = 82), LGIN (*n* = 60), HGIN (*n* = 64), ESCC (*n* = 70)	Characteristic in ESCC	China	Patients who had endoscopic and histological diagnosis	Subjects undergoing upper gastroenterology endoscopic examination because of screening for upper gastrointestinal cancer, then pathologically normal	Systemic diseases, use of antibiotics, proton pump inhibitors, prebiotics or other preparations in the previous month, or history of oral ulcers in the previous month	Endoscopic brushing (in the thoracic esophagus of approximately 20–38 cm from the central incisor)	16S rRNA gene amplicon sequencing (V4)	Ion S5TM XL (Thermo Scientific, Schwerte, Germany)	LEfSe method	*Granulicatella*, *Rothia*, *Streptococcus*, *Gemella*, *Leptotrichia*, and *Schaalia* were common biomarkers in patients with LGIN. *Lactobacillus* was a common biomarker in patients with HGIN, and *Bosea*, *Solobacterium*, *Gemella*, and *Peptostreptococcus* were common biomarkers in patients with ESCC. The functional composition of ESCC microbiota indicated a decreased nitrate reductase function.
Yang et al. ^84^	HC (*n* = 11), ESCC (*n* = 18)	Characteristic in ESCC	China	Patients who had endoscopic and histological diagnosis	Subjects with physiological normal esophagus	Use of antibiotics, hormones, intestinal flora regulator, or proton pump inhibitors, or with clinical infection	Endoscopic biopsies	16S rRNA gene amplicon sequencing (V4)	Illumina HiSeq 2500	LEfSe method, *t* test	*Fusobacterium* was more abundant, whereas *Klebsiella* was less contained in ESCC tissues than in HC. The functional composition of ESCC microbiota indicated decreased nitrate reductase functions and nitrite reductase functions compared with those in the HC.
Chen et al. ^85^	ESCC (*n* = 156)	Prognosis of ESCC	Taiwan	Patients with ESCC who received curative care	None	Not available	Endoscopic biopsies	Immunohistochemical detection, qPCR	None	Chi-square test, log-rank test, Cox proportional hazards regression model	*Porphyromonas gingivalis* was more abundant in ESCC tissues than in nontumor tissues. *Porphyromonas gingivalis* in ESCC tissues was correlated with advanced clinical stages and poor prognosis.
Li et al. ^86^	ESCC (*n* = 111)	Characteristic in ESCC	China	Patients with ESCC who underwent esophagectomy	None	Received preoperative radiotherapy and/or chemotherapy, or history of ESCC or inflammatory bowel disease	Surgical specimens	16S rRNA gene amplicon sequencing (V3–4), qPCR, whole-exome sequencing	Illumina MiSeq	*t* test	*Fusobacterium* and *Streptococcus* were more abundant, whereas *Butyrivibrio* and *Lactobacillus* were less contained in ESCC tissues than in nontumor tissues. The abundance of *Fusobacterium nucleatum* in ESCC tissues was correlated with clinical stages. Cell cycle, positive regulation of apoptotic process, positive regulation of transcription, DNA-templated, and the epidermal growth factor-related domain (EGF-like domain), further classified by the protein domain, were significantly enriched in ESCC tissues than in the nontumor tissues.
Jiang et al. ^87^	HC (*n* = 21), esophagitis (*n* = 15), ESCC (*n* = 32)	Characteristic in ESCC	China	Patients who had endoscopic and histological diagnosis	Subjects with normal esophagus confirmed by endoscopy and pathology and no digestive symptoms	Use of antibiotics, probiotics, proton pump inhibitor, or histamine 2 receptor antagonist within 1 month, or received radiotherapy, chemotherapy, and/or prior surgery	Endoscopic biopsies or surgical specimens	16S rRNA gene amplicon sequencing (V3–4)	Illumina MiSeq	LEfSe method	*Streptococcus* was more abundant, whereas *Faecalibacterium*, *Bacteroides*, *Curvibacter*, and *Blautia* were less contained in ESCC tissues than in the HC.
Lin et al. ^88^	ESCC (*n* = 120)	Characteristic in ESCC	China	Patients with ESCC who underwent esophagectomy, undergoing neither preoperative radiotherapy nor chemotherapy, and resident of the region for more than 10 years	None	Incomplete clinicopathological data and nonavailability of tissue samples, metastatic malignancy or recurrent EC, infection, or use of antibiotics, probiotics, or other drugs affecting the microbiota within 1 month	Surgical specimens (tumor and paired nontumor tissues from 3 cm distant)	16S rRNA gene amplicon sequencing (V3–4), qPCR	Illumina HiSeq 2500	analysis of composition of microbiomes 2 test	*Porphyromonas endodontalis* was more abundant, whereas *Helicobacter pylori* was less contained in ESCC tissues than in the adjacent nontumor tissues. Compared with that in tumor tissues, a denser and more complex association network was formed in adjacent nontumor tissues.
Shen et al. ^89^	ESCC (*n* = 19)	Characteristic in ESCC	China	Patients with ESCC who underwent esophagectomy	None	Received medical treatment within 1 month, or received chemotherapy or radiotherapy prior to the surgery	Surgical specimens (tumor and paired nontumor tissues from 2 cm distant)	16S rRNA gene amplicon sequencing (V1–9), qPCR	S/P2-C2/5.0 sequencing chemistry (Shanghai Biozeron Biotechnology Co. Ltd, Shanghai, China)	Wilcoxon rank-sum test	*Treponema* 2, *Lentimicrobiaceae*, *Selenomonas*, *Peptoanaerobacter*, and *Blautia* were more abundant, whereas *Methylobacterium* and *Akkermansia* were less contained in ESCC tissues than in the adjacent nontumor tissues. The complexity of microbial interactions in tumor tissues was weaker than that in the adjacent nontumor tissues. The LPS biosynthesis pathway of microorganisms in tumor tissues was more significant, whereas lipoic acid metabolism in the adjacent nontumor tissues was more prevalent.
Zhang B et al. ^90^	ESCC (*n* = 31)	Characteristic in ESCC	China	Patients with ESCC who underwent esophagectomy aged ≥50 yr	None	Received other antitumor therapy prior to surgery	Surgical specimens (tumor and paired nontumor tissues ≥5 cm distant)	16S rRNA gene amplicon sequencing (V3–4)	Illumina MiSeq	LEfSe method, *t* test	*Fusobacterium* was more abundant, whereas *Lactobacillus* was less contained in ESCC tissues than in the nontumor tissues. The abundances of *Treponema* and *Brevibacillus* in ESCC tissues were correlated with lymph node metastasis. The abundance of *Acinetobacter* in ESCC tissues was correlated with the tumor stages.
Ding et al. ^91^	ESCC (*n* = 73)	Characteristic in ESCC and prognosis of ESCC	China	Patients newly diagnosed with ESCC without treatment	None	Not available	Surgical specimens	qPCR	None	*t* test, log-rank test	*Fusobacterium nucleatum* was more abundant in ESCC tissues than in the adjacent nontumor tissues. *Fusobacterium nucleatum* was correlated with poor survival.
Lei et al. ^92^	ESCC (*n* = 181), EEC (*n* = 109), AEC (*n* = 72), HC (*n* = 10)	Characteristic in ESCC	China	Patients who had endoscopic and histological diagnosis	Not available	Not available	Endoscopic biopsies	qPCR	None	*t* test, Mann – Whitney *U* test, Chi-square test, Fisher’s exact test	*Lactobacillus*, *Escherichia shigella*, *Rikenellaceae-RC9*-gut-group, *Morganella*, and *Fusobacterium* were more abundant in the EEC of ESCC tissues than in the nontumor tissues. *Prevotella*, *Fusobacterium*, *Porphyromonas*, *Actinobacillus*, and *Neisseria* were more contained in the AEC of ESCC tissues than in the EEC.
Zhang JW et al. ^93^	ESCC (*n* = 107)	Characteristic in ESCC and prognosis of ESCC	China	Patients with ESCC who had received cisplatin -based neoadjuvant chemotherapy before surgical resection	None	Use of antibiotics within the 3 months, or other esophagus inflammatory diseases	Surgical specimens (tumor and paired nontumor tissues ≥5 cm distant)	16S rRNA gene amplicon sequencing (V3–4)	Illumina MiSeq	Mann – Whitney *U* test, log-rank test	*Fusobacterium*, *Peptostreptococcus*, *Porphyromonas*, *Prevotella*, and *Alloprevotella* were more abundant, whereas *Pseudomonas*, *Rhodococcus*, and *Achromobacter* were less contained in ESCC tissues than in the adjacent nontumor tissues. *Fusobacterium nucleatum* in ESCC tissues was correlated with lymph node metastasis and tumor TNM stage, shorter overall survival, and lower chemotherapy response rate.
Zhang S et al. ^94^	ESCC (*n* = 98)	Prognosis of ESCC	China	Patients with thoracic ESCC of histologic proof with complete follow-up data and complete surgical resection	None	Use of antibiotics or microecologics for at least 2 months, coexisting malignant tumors, preoperative neoadjuvant therapy, biotherapy, systemic inflammatory disease, or history of gastrointestinal surgery	Surgical specimens (tumor and paired nontumor tissues ≥3 cm distant)	16S rRNA gene amplicon sequencing (V4)	Illumina HiSeq	LEfSe method	The abundance of *Lactobacillus* was correlated with shorter survival after surgery.
Guo et al. ^95^	ESCC (*n* = 22)	Characteristic in ESCC and prognosis of ESCC	China	Patients newly diagnosed with ESCC without treatment	None	Use of antibiotics within 1 month, autoimmune, chronic and acute inflammatory diseases	Surgical specimens (no definition of paired nontumor tissue)	qPCR	None	*t* test	*Fusobacterium nucleatum* was more abundant in ESCC tissues than in the nontumor tissues.
Li et al. ^96^	ESCC (*n* = 19)	Characteristic in ESCC and prognosis of ESCC	China	Patients with ESCC who underwent ICIs	None	Not available	Surgical specimens (no definition of paired nontumor tissue)	qPCR	None	Mann – Whitney *U* test, *t* test, log-rank test	The abundance of *Fusobacterium nucleatum* and the PD-L1 protein levels were significantly higher in ESCC tissues than in the adjacent nontumor tissues. The higher abundance of *Fusobacterium nucleatum* in ESCC tissues was correlated with poor prognosis for ICIs.
Wu et al. ^97^	ESCC (*n* = 40)	Prognosis of ESCC	China	Patients with ESCC who underwent esophagectomy	None	Not available	Surgical specimens (no definition of paired nontumor tissue)	16S rRNA gene amplicon sequencing	Illumina MiSeq PE300	*t* test, log-rank test	The abundances of *Streptococcus* in ESCC tissues were correlated with favorable neoadjuvant chemoimmunotherapy response and disease-free survival.
Li et al. ^98^	^1^ ESCC (*n* = 52), HC (*n* = 52) ^2^ Normal (*n* = 9), esophagitis (*n* = 9), LGIN (*n* = 9), HGIN (*n* = 9), ESCC (*n* = 9)	Characteristic in ESCC	China	Patients who had endoscopic and histological diagnosis	Subjects detected as normal esophageal status based on endoscopic	Not available	Endoscopic brushing (five looping brushes at the lesion or normal middle esophagus)	16S rRNA gene amplicon sequencing (V4)	Illumina MiniSeq	LEfSe method, Kruskal – Wallis test	^1^*Fusobacterium*, *Prevotella*, and *Treponema* were more abundant, whereas *Actinobacillus*, *Haemophilus*, *Neisseria*, *Rothia*, *Streptococcus*, and *Veillonella* were less contained in ESCC tissues than in the HC. ^2^ *Fusobacterium nucleatum*, *Porphyromonas gingivalis*, and *Streptococcus australis* are potential candidates for early detection of the ESCC and precancerous lesions.
Shijimaya et al. ^99^	ESCC (*n* = 42), EAC (*n* = 3), non-EC controls (*n* = 20)	Characteristic in ESCC	Japan	Patients who had endoscopic and histological diagnosis	Subjects underwent upper gastroscopy for health check or secondary complete checkup of esophageal or stomach cancer followed by barium X-ray examination for the complaint of abdominal discomfort	Severe systemic disease, infection, malignancy in other organ, or use of antibiotics for 3 months	Endoscopic biopsies (Patients: EC tissues and their normal appearing esophageal mucosa), endoscopic wash samples (applied to the entire esophageal wall with no exclusive focus on areas that appeared abnormal)	16S rRNA gene amplicon sequencing (V3–4)	Illumina MiniSeq	Mann – Whitney *U* test	*Streptobacillus*, *Lachnoanaerobaculum*, *Leptotrichia*, *Peptococcus*, and *Moryella* were more abundant, whereas *Staphylococcus*, *Acinetobacter*, *Streptococcus*, and *Pseudomonas* were less contained in ESCC tissues than in the non-ESCC and non-EC controls. *Shuttlewarthia*, *Bergeryella*, and *Parvimonas* were more abundant, whereas *Prevotella*, *Campylobacter*, and *Anaerosinus* were less contained in ESCC wash samples than in the non-EC controls.
Sun et al. ^100^	ESCC (*n* = 28)	Characteristic in ESCC	China	Patients who had a histological diagnosis of ESCC	None	Receive chemotherapy, radiotherapy, or use of antibiotics for at least 6 months	Surgical specimens (tumor tissues and paired nontumor tissues at 5 cm distant)	qPCR, metabolomics and lipidomics analysis	None	*t* test, Chi-square test	*Fusobacterium periodonticum* was more contained in ESCC tissues than in the adjacent non-ESCC tissues. The *Fusobacterium periodonticum*-infected group was enriched in the top five metabolic pathways, primarily revolving around lipid metabolism, including steroid hormone biosynthesis, primary bile acid biosynthesis, unsaturated fatty acid biosynthesis, sphingolipid metabolism, and fatty acid elongation.
Wu et al. ^101^	ESCC (*n* = 24)	Prognosis of ESCC	China	Patients with locally advanced ESCC Treatment: Neoadjuvant chemoradiotherapy involved at least two cycles of platinum combined with paclitaxel chemotherapy and radiotherapy at least 40 Gy before curative esophagectomy and lymphadenectomy	None	Underwent non‐standard neoadjuvant therapy, including those who received only one cycle of chemotherapy	Endoscopic biopsies Sampling time points: before and after neoadjuvant chemoradiotherapy	16S rRNA gene amplicon sequencing (V3–4)	Illumina MiSeq PE300	LASSO logistic regression model	The higher abundance of *Prevotella* in ESCC tissues was in nonresponders, whereas *TM7x* and *Sphingobacterium* were in responders for neoadjuvant chemoradiotherapy.

AEC, advanced esophageal cancer; EEC, early-stage esophageal cancer; ESCC, esophageal squamous cell carcinoma; HC, healthy controls; HGIN, high-grade intraepithelial neoplasia; ICIs, immune checkpoint inhibitors; LEfSe, linear discriminant analysis effect size; LGIN, low-grade intraepithelial neoplasia.

**Figure 2.**
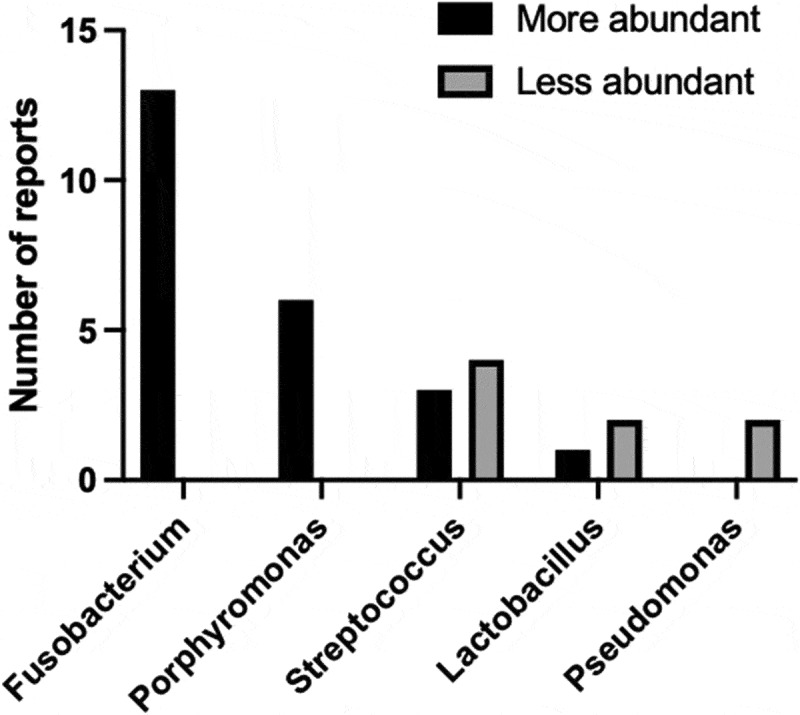
The bacterial genera reported to be more or less abundant in the esophageal microbiota of esophageal squamous cell carcinoma compared to nontumor tissues or healthy subjects.

The findings across the studies consistently showed that ESCC tissues exhibited altered esophageal microbiota profiles compared with non-ESSC or healthy tissues. This revealed that the number of specific bacterial genera or species either increased or decreased in each study.^61,76,77,79–93,93,95,98–100^ The bacterial genera that reported to be more or less abundant in the esophageal microbiota of ESCC compared to nontumor tissues or healthy subjects in two or more papers are summarized in Figure 2. In particular, across various studies, pathogenic bacteria such as *Fusobacterium*^77,79,80,84,86,90–93,95,96,98,100^ and *Porphyromonas*^76,81,85,88,93,100^ were consistently more abundant in ESCC tissues than in healthy tissues.

The bacterial genera reported in two or more papers are extracted. Details are described in Table 3.

The dynamic changes in the esophageal microbiota from intraepithelial neoplasia to ESCC can be potential biomarkers for monitoring disease progression.^81,83^ In addition, some bacteria were detected at different stages of ESCC progression.^76,77,79,80,85,86,90,92,93^ In particular, an increase in the number of *Fusobacterium nucleatum* (*F. nucleatum*)^77,79,80,86,93^ and *Porphyromonas gingivalis* (*P. gingivalis*)^76,85^ in the esophageal tumor microbiota in ESCC was more likely to be positively correlated with the progression of clinical stages.

Moreover, previous studies have revealed the utility of the esophageal tumor microbiota as a predictive biomarker of therapeutic response in patients with ESCC.^76,77,80,85,91,93,94,96,101^ In addition to disease progression, an increase in the number of *F. nucleatum*^77,80,91,93,96^ and *P. gingivalis*^76,85^ in the esophageal tumor microbiota was more likely to be related to poor prognosis in ESCC.

The dysbiosis of the esophageal microbiota, followed by alterations in the microbial cooccurrence network and host functional pathways in ESCC, may be involved in the carcinogenesis and progression of the local microenvironment for ESCC. Compared with the tumor tissue, a denser and more complex association network was formed in the adjacent non-tumor tissue of ESCC.^88,89^ Most differential abundances in adjacent non-tumor tissues were negatively associated with the epidermal growth factor receptor, erb-b2 receptor tyrosine kinase 2, erb-b2 receptor tyrosine kinase 4, and fibroblast growth factor receptor 1 signaling pathways. Further, they were positively associated with the MET protooncogene, receptor tyrosine kinase, and phosphatase and tensin homolog signaling pathways.^88^ The lipoic acid metabolism in adjacent non-tumor tissues was more prevalent. Meanwhile, the LPS biosynthesis pathway of microorganisms in tumor tissues was more significant.^89^ In the ESCC tissues, the functional composition of the ESCC microbiota had decreased nitrate reductase functions and nitrite reductase functions compared with the healthy tissues.^83,84^ In addition, via whole-exome sequencing and functional metagenome prediction, the cell cycle, positive regulation of the apoptotic process and transcription, DNA-templated synthesis, and further classification of the epidermal growth factor-related domain (EGF-like domain) by protein domain were significantly enriched in the ESCC tissues than in the non-tumor tissues.

The abundance of *F. nucleatum* and *P. gingivalis* was more likely to increase in ESCC tissues. Moreover, it was positively correlated with progression and poor prognosis in patients with ESCC. The possible mechanisms associated with the pathogenesis and progression of ESCC due to these processes have been reported.

*P. gingivalis* infection increased interleukin IL-6 levels and it promoted the epithelial–mesenchymal transition and myeloid-derived suppressor cells recruitment.^85^ *P. gingivalis* inhibited epithelial cell apoptosis and promoted carcinogenesis through the activation of PI3K/Akt signaling^102,103^ and the inhabition of P2X7^104,105^ and other pathways.^106^ In recent study, on a transcriptomic analysis, the presence of a *P. gingivalis* infection increased transforming growth factor-beta (TGF-β) levels and enhanced predominantly Glycoprotein A expression repetitions, thereby activating TGF-β/Smad signaling.^107^ The *P. gingivalis* infection remarkably activated TGF-β signaling and subsequently enhanced the tumor growth and metastasis in xenograft models.^107^ Thus, *P. gingivalis* is thought to be implicated in the pathogenesis and progression of ESCC.

Previous studies have reported some mechanisms associated with the effect of *F. nucleatum* on the pathogenesis, progression, and chemoresistance of ESCC. *F. nucleatum* promoted ESCC progression via the NOD1/RIPK2/NF-κB pathway.^108^ Moreover, *F. nucleatum* promoted early ESCC development by upregulating the IL-32/PRTN3 expression and activating the PI3K/AKT signaling pathway.^92^ *F. nucleatum* accelerated cell proliferation by activating the AHR/CYP1A1 signaling pathway in ESCC.^109^ Intracellular *F. nucleatum* infection increases METTL3 transcription. Then, METTL3 promotes c-Myc mRNA methylation in the 3′-untranslated region and enhances its mRNA stability in a YTH N6-Methyladenosine RNA binding protein 1-dependent manner, which contributes to *F. nucleatum*-induced proliferation and metastasis in ESCC.^95^ *F. nucleatum* inhibits the proliferation and cytokine secretion of T cells, and Fn-Dps binds to the PD-L1 gene promoter that activates transcription factor-3 to transcriptionally upregulate PD-L1 expression.^96^ In terms of chemoresistance, *F. nucleatum* increased the expression of NLRP3 and then enriched the expression of myeloid-derived suppressor cells, thereby leading to cell chemoresistance in ESCC.^110^ In addition, *F. nucleatum* invaded and survived in senescent ESCC cells. This mechanism further activates the DNA damage response pathway, which enhances the senescence-associated secretory phenotype and promotes ESCC recurrence and chemotherapy resistance.^93^ These associated mechanisms are being recognized. In particular, *F. nucleatum* is the most commonly implicated in the pathogenesis and progression of ESCC.

Based on recent research, ESCC tissues exhibited a significant higher abundance of *Fusobacterium periodonticum* (*F. periodonticum*) than adjacent non-tumor tissues.^100^ According to a metabolomics and lipidomics analysis, in the *F. periodonticum*-infected group, the enriched metabolic pathways primarily revolve around lipid metabolism.^100^ In ESCC, the N-nitrosamine-mediated upregulation of FadAL induces the formation of epithelial mesenchymal transition (EMT) subtypes by directly interacting with FLOT1 and promoting palmitic acid accumulation, thereby enhancing Wnt3A palmitoylation and activating the Wnt/β-catenin signaling pathway.^100^ Regarding mechanisms, *F. periodonticum* alters metabolic profiles, enhances glycolysis, and releases lactic acid, which is regulated by the TLR4/Akt/HIF-1α signaling pathway.^111^ Then, lactic acid promotes EMT in ESCC and enhances migratory and invasive capabilities via the GPR81/Wnt/β-catenin signaling pathway.^111^ Thus, *F. periodonticum* may promote EMT in ESCC, and it can be a future treatment target.

### Stomach microbiota and its association with esophageal cancer

16S rRNA sequencing reveals a diverse stomach microbiota community.^112^ However, most EC studies have focused on the association between EC and *Helicobacter pylori* (*H. pylori*).

Several studies have shown an inverse association between *H. pylori* infection and EAC.^113–120^ These epidemiologic evidence indicates that *H. pylori* infection is a protective factor against EAC development. This can be attributed to the fact that *H. pylori* may suppress gastric acid secretion, reduce the risk of developing GERD and BE, and decrease the EAC cascade. In contrast, *H. pylori* infection in esophageal epithelial cells has a detrimental effect, leading to the dysregulation of micro RNAs and subsequently causing the overexpression of intestinal metaplasia factors and carcinogenic factors such as caudal-type homeobox 2 and cyclooxygenase-2.^121^ The association between *H. pylori* and EAC is still controversial.

Currently, there is no definite evidence showing that *H. pylori* infection contributes or inhibits the incidence of ESCC. A large meta-analysis revealed no significant associations between ESCC and *H. pylori* infection.^113,122^ A recent report with a small number of cases showed that the tumor microenvironment in ESCC with *H. pylori* infection exhibited an immunosuppressive phenotype.^123^ Thus, *H. pylori* may influence the tumor microenvironment in ESCC. However, there is no sufficient evidence supporting this notion.

Moreover, there are a few reports on the association between stomach microbiota and EC, in addition to *H. pylori*, based on an analysis using endoscopic gastric mucosa samples. The stomach microbiota composition of the gastric fundal mucosa differs between patients with early ESCC and ESD and those with a normal esophagus, with a higher abundance of bacteria in the orders *Clostridiales* and *Erysipelotrichales*.^124^ The Firmicutes/Bacteroidetes ratio of the early-stage intramucosal ESCC group was significantly lower than that of the normal esophagus group. This indicated the potential dysbiosis of stomach microbiota in patients with ESCC.^125^

In summary, epidemiological reports have shown that *H. pylo*ri infection is inversely associated with the development of EAC, but not with ESCC. However, this associated is not yet established, and the association between stomach microbiota and EC remains unclear.

### Future perspectives on the role of intestinal and esophageal microbiota in esophageal cancer

The impact of intestinal and esophageal microbiota on the development and progression of EC is becoming clearer. However, the results of current studies vary. The use of intestinal and esophageal microbiota in the clinical assessment of EC is till challenging.

The early detection of EC using fecal samples, which are noninvasive and simple to use, is a promising clinical modality for EC. However, the intestinal microbiota profiles in fecal samples that characterize EC are inconsistent. Hence, they are still challenging to use for the early detection of EC. The intestinal microbiota in fecal samples has been used for the early detection of various types of cancers, but it has not yet been clinically applied. For example, in colorectal cancer, which is considered as closest to clinical applications, metagenomic and metabolomic markers are used to differentiate patients with intramucosal carcinoma from healthy ones. The relative abundance of *F. nucleatum* is significantly, continuously elevated in intramucosal carcinoma to more advanced stages.^126,127^ Moreover, the number of *Atopobium parvulum* and *Actinomyces odontolyticus* are significantly high only in multiple polypoid adenomas and/or intramucosal carcinomas.^126^ Moreover, based on metabolome analyses, the expressions of branched-chain amino acids and phenylalanine in intramucosal carcinomas and bile acids, including deoxycholate, were significantly high in multiple polypoid adenomas and/or intramucosal carcinomas.^126^ Based on these results, the early diagnosis of intramucosal carcinoma is being attempted by extracting DNA from stools, examining bacterial-derived genes using whole-genome shotgun sequencing, and combining several bacterial species and metabolites for machine learning.^128^ However, such a strategy that uses intestinal bacteria for the early detection of colorectal cancer has not been used in clinical practice.

In clinical settings, the intestinal and esophageal microbiota are most likely to be applied as a biomarker for predicting the efficacy and adverse events of treatment in EC. Several clinical studies have revealed that specific intestinal microbiota such as *Akkermansia muciniphila*^129–134^ and *Faecalibacterium prausnitzii*^135–139^ can be biomarkers for predicting the efficacy of ICIs. Nevertheless, the use of these intestinal microbiota as biomarkers for predicting the efficacy of immune checkpoint inhibitors in EC should be validated in a large cohort. Previous research has revealed the association between intestinal bacteria and a series of immune-related adverse events during ICIs administration in patients with gastrointestinal cancers, including EC.^41,140^ Further, *F. nucleatum* in the esophageal tumor microbiota can be promising for prognostic prediction in ESCC.^77,80,91,93,96^ Thus, the clinical application of the intestinal and esophageal microbiota as a biomarker for predicting treatment efficacy and adverse events is important for promoting individualized treatment.

In the future, combining conventional therapy and probiotics, prebiotics, and synbiotics is the next step in the clinical application of intestinal microbiota as a novel strategy to improve the success rate of EC treatment. In EC, probiotics, prebiotics, and synbiotics have been used in the past. However, they aim to prevent and improve complications from surgery and anticancer drugs.^141–146^ Recently, there have been attempts to induce the intestinal microbiota and their metabolites to increase antitumor effects. In laboratory studies, the probiotics *Lactobacillus rhamnosus*,^147^ *L. acidophilus*, *L. plantarum*, *L. fermentum*,^148^ and *Lactobacillus casei*^149^ and the prebiotic cranberry proanthocyanidins^150^ in vivo and in vitro have been found to have antitumor effects against EC. *L. rhamnosus* has antitumor effects by reducing the expression of the Wnt signaling pathway genes.^147^ Supplementation with Lactobacillus species after bile injury accelerated the repair of bile-induced DNA damage via the recruitment of pH2AX/RAD51. Furthermore, it reduced NF-κB-associated inflammation in esophageal cells.^148^ Ferrichrome, produced by the probiotic *Lactobacillus casei*, induces DNA damage-inducible transcript-3, thereby producing antitumor effects, including cell cycle arrest and apoptosis in EC cells.^149^ Cranberry proanthocyanidins exhibit prebiotic activity by abrogating GERD-induced dysbiosis of the intestinal microbiota and mitigating bile acid metabolism and transport. This results in the significant inhibition of EAC via TLR/NF-κB/TP53 signaling cascades.^150^

Further, to modulate the intestinal microbiota composition in humans, fecal microbiota transplantation (FMT) is one of the strategies that are now being investigated. In EC, FMT from a healthy donor prior to first-line chemotherapy improved the response and survival of patients with metastatic EC.^151^ In melanoma treated with ICIs, FMT shifted the intestinal microbiota composition toward a favorable ICIs efficacy to induce clinical responses.^152,153^ In addition, better disease control rate was achieved with the combined use of ICIs and FMT for advanced gastrointestinal cancers.^154^ Clinical studies on combination therapy with ICI and FMT for patients with unresectable advanced-stage or recurrent EC are ongoing.^155^

Several studies have shown that *F. nucleatum* functions as an oncogenic bacterium. Moreover, it is implicated in the pathogenesis and progression of ESCC. *F. nucleatum*, which is the bacterium in the esophageal tumor microbiota, itself or its metabolic products and regulated pathways can be therapeutic targets.

Although an association was observed between the intestinal and esophageal microbiota and EC, whether the association is causal or merely correlational is not yet completely understood. For example, whether changes in the intestinal and esophageal microbiota contribute to the development and progression of EC or whether EC alters their composition has not been confirmed. In addition, different methods can be used for sampling and analyzing samples in the study of intestinal bacteria, and it is difficult to reproduce or compare results. Thus, a standardized protocol should be established. Further, the characteristics of the intestinal and esophageal microbiota are significantly influenced by different factors including diet, medications, age, genetics, ethnicity, and lifestyle habits. Further detailed studies must be performed to validate the application of the intestinal and esophageal microbiota of EC in clinical settings.

## Conclusion

Patients with EC worldwide have a poor prognosis. The association between EC and the intestinal and esophageal microbiota should be further elucidated to improve the efficacy of treatment modalities against EC. The role of dysbiosis of the intestinal and esophageal microbiota in the development, progression, and treatment of EC is becoming clear. *F. nucleatum* functions as an oncogenic bacteria, and it is implicated in the pathogenesis and progression of ESCC. In terms of potential, it is most suitable as a biomarker for predicting therapeutic efficacy in clinical settings. Further, combining conventional therapy and probiotics, prebiotics, and synbiotics or FMT may improve therapeutic efficacy in EC. Nonetheless, the clinical application of this novel combination therapy has several issues. The characteristics of the intestinal and esophageal microbiota are significantly influenced by several factors including diet, medications, age, genetics, ethnicity, and lifestyle habits. Therefore, due to significant individual variations, the application of the intestinal and esophageal microbiota as a biomarker of EC in clinical practice is still challenging. The attempt to examining bacterial-derived genes using whole-genome shotgun sequencing and combining several bacterial species and metabolites for machine learning may be a promising new approach. Further, more detailed studies on the adaptation of the intestinal and esophageal bacteria to EC treatment should be performed to facilitate individualized treatments.

## Materials and methods

This review for the impact of the intestinal and esophageal microbiota on EC (ESCC or EAC) development and treatment involved a systematic literature search and data extraction process.

### Systematic search to identify studies for inclusion

On February 21, 2025, we conducted a systematic literature search for published research articles using MEDLINE/PubMed using the following search terms: ((((esophagus cancer) OR (esophageal cancer)) OR (esophagus neoplasm)) OR (esophageal neoplasm)) AND ((((((((gut microbes) OR (gut microbiota)) OR (intestinal microbiota)) OR (microbiota)) OR (microbiome)) OR (microbial)) OR (gastrointestinal microbiota)) OR (esophageal microbiota)). The outcomes of interest were: (1) differences in intestinal or esophageal microbiota composition between EC patients and healthy control group; (2) differences in esophageal microbiota composition between EC tissue and non-EC tissue; (3) associations between intestinal or esophageal microbiota composition and prognosis of EC.

### Identification of primary studies for inclusion

Inclusion criteria were: (1) human observational studies; (2) patients with a diagnosis of EC; (3) Outcome indicators including the quantitative abundance of gut microbiota.

Exclusion criteria were: (1) only cell studies and animal studies; (2) intervention studies; (3) multiple types of cancer are analyzed together and there is no analysis of EC separately; (4) analysis of existing publicly available databases only; (5) review articles, letters to the editor, editorial comment, and case reports; (6) studies with incomplete or unreported data; (7) written in a language other than English.

Two reviewers (YB and KT) independently screened titles and abstracts for relevant papers, with conflicts discussed to achieve consensus on inclusion and exclusion of retrieved titles.

### Study selection and extraction

A total of 611 studies were retrieved, and we extracted studies according to inclusion and exclusion criteria. Furthermore, re-extraction from these studies’ reference lists and Google Scholar citation checks based on the criteria. Finally, were finally included: (1) 17 for relationship between dysbiosis of intestinal microbiota and EC; (2) 11 for relationship between esophageal microbiota and EAC; (3) 27 for relationship between esophageal microbiota and ESCC.

The extracted studies are summarized in Tables 1–3.
